# Knowledge, Attitudes, and Beliefs About Colorectal Cancer Screening in Puerto Rico

**DOI:** 10.1007/s13187-022-02153-z

**Published:** 2022-03-31

**Authors:** Vivian Colón-López, Ileska M. Valencia-Torres, Elsa I. Ríos, Josheili Llavona, Camille Vélez-Álamo, María E. Fernández

**Affiliations:** 1 Division of Population Health Sciences, PR Comprehensive Cancer Center, Medical , University of Puerto Rico, Sciences Campus, PMB 371, P.O. Box 70344, San Juan, PR 00936-5067 USA; 2 Health Services Administration, Evaluation Program, Graduate School of Public Health, University of Puerto Rico, PMB 371, P.O. Box 70344, San Juan, PR 00936-5067 USA; 3 Center for Health Promotion and Prevention Research, The University of Texas School of Public Health, 7000 Fannin St., Suite 2080, Houston, TX 77030 USA; 4 UPR-MDACC Partnership for Excellence in Cancer Research Program, University of Puerto Rico, PMB 371, P.O. Box 70344, San Juan, PR 00936-5067 USA

**Keywords:** CRC screening, Focus groups, Federally qualified health centers, Barriers and facilitators, Latinos/Hispanics

## Abstract

The purpose of this study was to describe the psychosocial factors influencing participation in colorectal cancer screening (CRCS) among Puerto Rican men and women. We conducted seven focus groups in metropolitan and rural areas of Puerto Rico (PR) with men and women (using gender specific groups) aged 50 to 80 years (*n* = 51) who were non-adherent to CRC guidelines. The focus group guide included questions related to colorectal cancer (CRC) and CRC screening knowledge, attitudes, and beliefs. We analyzed data using a modified grounded theory approach to identify emergent themes. Focus groups revealed seven major themes that represented barriers to CRCS: (1) lack of CRC knowledge, (2) lack of knowledge about colorectal cancer screening tests as well as the required preparation, (3) embarrassment, (4) low perceived benefit of CRCS and sense of fatalism, (5) transportation (mostly among participants in rural areas), (6) lack of time, and (7) financial burden. All participants understood the benefits of CRCS once the procedure was explained. Additionally, participants reported a lack of provider recommendation for CRCS. In this group of Puerto Rican participants who were non-adherent to CRCS, there were misconceptions about CRC, screening tests available, and preparation and testing procedures. Participants’ low levels of knowledge and negative attitudes concerning CRCS and low reported provider recommendation were important deterrents to screening. These findings suggest the need for educational efforts to increase knowledge and attitudes about CRCS and improved patient-provider communication to reduce missed opportunities to recommend.

**Supplementary Information:**

The online version contains supplementary material available at 10.1007/s13187-022-02153-z.

## Introduction

Colorectal cancer (CRC) is the leading cause of cancer death in Puerto Rico and third among Hispanics in the USA [1]. The age-adjusted incidence rate in Puerto Rico (PR) by county between 2012 and 2016 was 51.6 per 100,000 men and 34.8 per 100,000 women. Local data show that only 39.9% of CRC patients were diagnosed with localized-stage CRC, while 33.6% were diagnosed with regional-stage CRC, and 15.4% with distant stage CRC (Alvarado-Ortiz, personal communication, May, 2020). Patients diagnosed with a localized stage have a 5-year survival rate of 90%, while survival declines to 70% and 13% for patients diagnosed with regional and distant stages, respectively [2]. Thus, improvements in early detection and prevention through detection of precancerous changes is critically important to reduce the CRC burden in Puerto Rico.

CRC screening through fecal occult blood test (FOBT), colonoscopy, or sigmoidoscopy can help prevent CRC or detect it in earlier stages, when treatment is most effective. The US Preventive Services Task Force (USPSTF) recommends colorectal cancer screening (CRCS) for all adults 50 to 75 years old [3]. The National Colorectal Cancer Roundtable (NCCRT) launched an initiative aiming to increase the use of recommended CRCS tests among the entire population for whom screening is appropriate. The goal is to have 80% of adults age 50 and older regularly screened for CRC in every community. However, CRCS rates in PR have lagged behind these goals. Data from the Behavioral Risk Factor Surveillance System [4] indicates that only 9.5% of adults 50–75 years of age living in PR have had an FOBT within the past year and 52.3% had a colonoscopy in the past ten years. Given the high CRC mortality rates in PR and low screening, it is important to better understand why screening rates are so low and to develop interventions to increase screening and follow-up care. Due to the documented burden of CRC on the island, we planned and developed a health promotion intervention to increase CRCS among men and women attending Federally Qualified Health Clinics (FQHCs) in PR.

In 2015, we initiated efforts to develop an educational program for colorectal cancer prevention [5]. With this initiative, collaborative efforts between us, the Puerto Rico Primary Care Association (PRPCA), and FQHCs were established to use those settings as community implementation laboratories in the delivery of our program. FQHCs are non-profit, community-based corporations that provide primary and preventive health care. In PR, these clinics serve communities with limited resources and barriers to medical care by facilitating access to services regardless of the patient’s ability to pay. In 2014, these centers, also known as Primary Health Centers, offered preventive and primary services to 330,736 patients.

We used intervention mapping (IM), a systematic framework using theory and evidence [6] to plan a health promotion interventions to develop our program. The goal was to increase colorectal cancer screening (CRCS) among Puerto Rican adults 50 years and older who are patients of FQHCs in PR. We conducted a need and asset assessment including the conduct of a qualitative study to inform the development of the intervention and the results of that study are presented here.

## Methods

We conducted seven focus groups to identify community needs and resources, specific sub-behaviors related to CRCS (performance objectives), and the determinants of CRCS [5]. We recruited patients from three different sites: HealthProMed, Castañer General Hospital, and Casa Castañer. HealthProMed is one of nine FQHCs in the San Juan metropolitan area. It is located in a primarily urban area comprising San Juan and surrounding cities. The second site (Castañer General Hospital) is an institution founded in 1942 located in the mountains between the rural municipalities of Lares, Adjuntas, Maricao, Yauco, and Las Marías. In 2014, Castañer General Hospital provided services to 10,358 patients, of whom 10–25% were 50 years of age or older. Additionally, we recruited patients from Casa Castañer, an elderly house which is not a primary health center, but it is located at a walking distance from the Castañer General Hospital. We used this location to facilitate the participation of residents who were also patients at Castañer General Hospital. Between February and April 2014, we conducted seven focus groups. Three focus groups were conducted at HealthProMed (two with women and one with men) and, of the remaining four focus groups, two were conducted at the Castañer General Hospital and two at Casa Castañer (one with women and one with men in each site).

### Recruitment and Data Collection

The inclusion criteria for the focus groups were being 50 years or older, with no prior history of CRC, and not being up to date with CRCS (not having completed a fecal occult blood test -FOBT or FIT- in the last year, no sigmoidoscopy in the past 5 years, nor a colonoscopy in the past 10 years (or in the past 5 years for those participants with a family history of CRC). Participants were recruited in the FQHCs waiting rooms and surrounding areas using an eligibility form. Once recruited, potential candidates were informed of the day and location where the focus groups would take place. Each focus group lasted approximately 90 min.

### Interview Guide and Demographic Survey

Our research team and collaborating members of the Colorectal Cancer Coalition of PR adapted an interview guide from a previous study [7]. The guide included questions exploring knowledge, attitudes, and beliefs about CRC, CRCS, and early detection tests barriers and facilitators (Table 1), as well as brief explanations of CRC and CRC screening methods (Table 2). Participants also responded to a short demographic questionnaire.

**Table 1:** Interview questions from focus groups conducted in Puerto Rico with men and women between the ages of 50 to 80, about their knowledge, attitudes, and beliefs regarding the screen process leading to the early detection of colorectal cancer

Topic	Questions
Awareness and knowledge about cancer colorectal cancer and prevention	Have you heard about colorectal cancer or colon cancer? What do you think is colorectal cancer? What have you heard about colorectal cancer? Who can get colorectal cancer? Do you think colorectal cancer affects more than one group of people (men, women, and the elderly)? Do you think that you could ever have this disease in your life? Why? Why not? What are some ways to prevent colorectal cancer?
Awareness and knowledge about cancer screening test	Could you name an early detection cancer-screening test? Could you explain what do you know about the test? Can you describe it? Who should be tested? How often do you think people of your age should be screened with this test? Have you been screened for colorectal cancer? Who recommended it (the screening test) and why? What kind of test was recommended?
Awareness and knowledge about FOBT	What have you heard about the Fecal Occult Blood Test in feces or FOBT? Who told you about FOBT? Have you or someone you know been screened with a FOBT? If a doctor recommends you having a FOBT as an early screening test for colorectal cancer, would you do it? What are some things that would make it difficult for you to do the FOBT? What information would you like to know about this test before you decide to do it?
Awareness and knowledge about colonoscopy	What have you heard about colonoscopy? Has a doctor recommended you the colonoscopy for colorectal cancer (CRC) screening? If a doctor recommends you to have a colonoscopy as an early screening test for colorectal cancer, would you do it? What things may make it difficult for you to have a colonoscopy performed?
For never-screened people: attitudes toward CRC screening test	Have you ever thought about being screened for CRC? Can you talk about some of the reasons why you have never been screened for CRC? What things would help you to be tested? If you want to be screened for colorectal cancer today, what would you do to: (A) Make transportation arrangements? (B) Manage the barriers that prevent you from being screened? (C) Handle problems with the health insurance plan or medical referral? (D) Decide to be screened? (E) Work with the possible fears that prevent you from being screened?
Communication with PCP	How would you describe your relationship with your primary care provider? Does your doctor talk to you about early screening tests for people your age? Can you describe your experience with referrals for diagnostic tests or early detection tests?
Recommendations about educational materials	Which information regarding colorectal cancer and early detection tests presented in these educational materials do you understand are important? How would you like to see this material presented? In what format would you like to have this information?

**Table 2:** Concept definitions or explanations provided to focus group participants

Concept	Definition/Explanation
Colorectal cancer	Colorectal cancer is a disease in which malignant (cancer) cells grow in the tissues of the colon or rectum
Fecal Occult Blood Test (FOBT)	The fecal occult blood test (FOBT) is used to find hidden blood (blood that cannot be seen with the naked eye) in the stool
Colonoscopy	During the colonoscopy, the physician looks at the entire colon and rectum with a colonoscope. It is introduced through the rectum to the colon. The colonoscope has a video camera at its end that is connected to a monitor so that the physician can closely observe and examine the inside of the colon. Through the colonoscope, special instruments can be passed to perform a biopsy or to remove any suspicious areas, such as polyps, if necessary. During the colonoscopy, they will inject medicine into your arm to make you sleepy. Afterwards you will need someone to drive you home and you may need to take the rest of the day off for not being able to do your daily tasks. It is recommended to have a colonoscopy every 10 years
Sigmoidoscopy	During the sigmoidoscopy, the physician observes and examines the lower part of the colon using a lighted flexible tube about the thickness of a finger and which has a small video camera on the end. It is introduced through the rectum, the images obtained from the endoscope are observed on a monitor. During the sigmoidoscopy, you stay awake, can drive your car home, and can resume normal activities. It is recommended to have it performed every 5 years

### Focus Group Discussion

The number of participants for each focus group varied from four to twelve. After the research staff explained the study purpose, each participant was given an informed consent form. Then, participants completed a survey that included information regarding history of CRCS testing. The research team moderated, recorded, and took notes during of each focus interview group. After the focus group, participants received CRC and CRCS educational materials, along with $20 as compensation for their time.

During the focus group, the moderator first used the focus group guide to elicit open-ended discussion. Once participants responded to questions regarding CRC and CRCS, the moderator provided a brief explanation about CRC, the Fecal Occult Blood Test (FOBT), the colonoscopy, and sigmoidoscopy (Table 2) in order to provide a general understanding to all participants. After the brief explanation, the moderator initiated a second discussion round with the participants to assess their perspectives on the previously discussed topics after having received the additional information provided by the moderator.

### Data Management and Analysis

Focus groups were audio-recorded and transcribed. Then, an excel spreadsheet was created for indexing and summarizing data. All transcripts were read and coded by the lead and second author using a constant comparison method consistent with a grounded theory approach [8]. Coders read and coded the same transcript, discussed identified themes (Table 3), and created a dataset of codes including all transcripts. The team met biweekly to discuss new themes and to come to consensus on new codes and coding. IBM SPSS 19 statistical package was used to analyze quantitative data to provide a descriptive profile of study participants.

**Table 3:** Themes and sub-themes

Themes	Sub-themes
Health behaviors	Lifestyle and risk factors for CRC
Family health history	Family history of cancer, colon or rectal polyps, and CRC
Colorectal cancer screening behaviors	
Internal factors influencing colorectal cancer screening	Knowledge • Limited knowledge about CRCS • Knowledge about CRC • Family/friends experience with cancer or screening • Information needed/wanted • Experiences with CRCS • Limited knowledge about cancer screening • Knowledge about cancer screening • Experiences with cancer screening • Limited knowledge about cancer
	Attitudes/Beliefs • Fear of cancer • Perceived risk of CRC • Perceives benefits • Outcome expectations • Spirituality/Religion • Death • Fatalism Low perceived risk for cancer
	Perceived Facilitators • Willingness to have CRCS • No barriers for CRCS • Good experiences with cancer screening
	Perceived barriers for screening • Fear/uncomfortable feelings about CRCS • Bad/uncomfortable experiences with cancer screening • Fear of cancer screening
External factors influencing colorectal cancer screening	System level barriers Lack of Access to health services • Transportation barriers • Personal barriers to CRCS • Financial burden for CRCS Lack of communication with Primary Care Provider (PCP) • PCP has not recommended or discussed CRCS • Poor or lack of a personal relationship with PCP System level facilitators Communication with PCP and family • PCP has recommended or discussed CRCS • Good personal relationship with PCP • Family/peer support

### Findings

A total of 51 individuals participated in the focus groups, 50 of whom completed the survey. Sociodemographic characteristics of the focus groups participants are summarized in Online Resource 1. The mean age of the participants was 61.9 ± 6.8 years. More than half of the participants (56%) were female and more than one-third (36%) were married or living with a partner. More than three quarters of the participants had a high school education or less (77.1%), were unemployed (76%), and were insured by the Government Health Plan (72%). The majority (87.8%) of the participants had an annual household income of less than $15,000. Information regarding participants’ screening and other health behavior indicators related to CRCS are shown in Online Resource 2. More than have of our participants (54.6%) reported smoking and less than a quarter of the participants (22%) reported having had a FOBT in their lifetime; only 6% had ever had a colonoscopy.

### Factors Influencing CRC Screening Behavior

#### Knowledge

Participants were asked what they knew about CRC (“Have you heard about colorectal cancer or colon cancer? What do you think is colorectal cancer? What have you heard about colorectal cancer?”) and CRCS (“What are some ways to detect colorectal cancer early? Have you been recommended for colorectal cancer screening?”). Men and women from both urban and rural settings demonstrated having very limited knowledge about CRC and CRCS. Those who had some knowledge had learned about CRC and CRCS from TV ads, educational sessions at their clinics, or their physicians. CRC knowledge was limited to understanding some symptoms (rural region man: “[CRC] are polyps”; urban region woman: “…there is bleeding”.), and the age group affected by it (“the elderly”; “those over 50”). Though participants (regardless of their group, urban or rural) had some understanding about certain factors associated with CRC, they admitted that they did not understand it thoroughly, nor were they able to explain what it entails. A man from the urban clinic said: “Look, I understand a little about CRC. I will not be able to answer your question, because I understand just a little…" A woman from the same clinic commented: “I have not heard much.”

Participants in both settings indicated that they knew there were CRCS methods available; but just like with CRC, they were not able to describe the test accurately. Some participants mentioned that they did not know about screening tests for CRC; in some cases, they mentioned other laboratory tests (“blood tests”), but did not identify the type of test. When asked to describe those “blood tests,” some were able to describe the procedures for the FOBT (urban setting man: “It is a test to see if there is blood in your stool; to detect bleeding”). Very few participants mentioned the colonoscopy as a CRCS test.

#### Responsiveness to Information

Following the discussion about what they knew, the moderator provided a brief explanation about CRC and CRCS and participants were asked once more about their understanding of each topic. When asked about the colonoscopy, participants indicated that before the moderator explained the procedure, they were not aware about what it entailed or how often it should be performed. A woman from the urban area mentioned: “I truly don’t know much other than the doctor telling me that I have to get screened. Well, I have heard about [the colonoscopy] but not as detailed as now. But for example, I knew that it was a test that is [performed] in the colon; but [the process of the colonoscopy], I had not heard [before].” Most participants indicated how important it was for them to be informed about test procedures before having them done. A participant from the urban region explained that he needs this type of information because it would allow him to be prepared: “[…] you go prepared; with the mind set on it. You’re going to be scared, but you already know what is going to happen to you.” A woman who attends the rural clinic commented about the FOBT: “I want to know, they have to explain to me why they are going to do it [FOBT], since I do not know.”

#### Experiences with CRC Screening

Those participants who had heard of CRCS indicated having heard about the FOBT or the colonoscopy from a family member who had been screened or because a physician had recommended the test. However, some participants who had received a recommendation admitted not having followed through with the recommendation. Some of those who had been screened shared that they did because a spouse or family member recommended doing so. A man who attends the rural clinic described his experience with the colonoscopy: “[…] I got screened because I was almost forced [by my wife].” Others indicated having received a diagnostic colonoscopy because of symptoms or illness, not as a preventive measure. A man from the urban setting shared: “I went through the colonoscopy test when I had a disease. They did it, I was injected to sleep but I never slept, they were waiting for me to fall asleep, but I did not fall asleep. They have a TV so you can see how you are inside; I saw everything.”

### Attitudes/Beliefs

#### Low Perceived Benefit of CRCS and Sense of Fatalism

When participants were asked if they knew about any risk factors for CRC, participants mentioned age, high-fat diet, alcohol consumption, and smoking. During the group discussion, participants acknowledged they could be at risk for CRC mostly due to their age and unhealthy dietary practices. Nonetheless, both women and men indicated they did not consider it necessary to go to the doctor if they do not have any symptoms (“I don’t go to the doctor because I feel well.”) or if they had previous screenings with no abnormal results. A woman from the rural setting said: “… every year the doctor provides me with the referral for the [screening] tests; but they always came back normal so I haven’t been back to the doctor in 12 years.” However, they also expressed that even if they might have symptoms, they still delayed getting screened. Participants believe that being screened for CRCS or cancer in general, when having symptoms will most probably result in an abnormal screening, which would finally result in death. Both men and women conveyed this sense of fear and fatalism. A man in the rural setting said that he did not get screened for colorectal cancer because he is fearful of the diagnosis: “…fear of being told that I have cancer.” A woman from the urban setting expressed: “[Having] Colorectal cancer is very dangerous. If you do not get screened early on when you finally do, as they say, you have been ‘taken’ by it.”

### Perceived Barriers for Screening

#### Fear/Uncomfortable Feelings About CRC Screening

Some participants shared not feeling comfortable with the screening tests for CRC. Fear of the screening process, discomfort with the special diet in preparation for the test, feeling embarrassed by the colonoscopy procedure, and trepidation of a possible cancer diagnosis are some of the personal barriers mentioned. A man from the rural area shared: “…some of my friends sometimes tell me things that make me afraid of [the procedure]. They tell me that it hurts… Believe me … I have not had it done out of fear.” Both men and women said that embarrassment was one reason they had not gotten screened. A man from the rural area shared: “Well look, this, I have not done it because, hmm… I have felt a little embarrassed if you want me to tell you, to be honest. I have felt a little embarrassed, and I have not wanted…” A woman from the rural area said, “Embarrassment is always there, as I say… I think I have to emotionally prepare myself more than physically.”

Various participants discussed how complying with the diet before the colonoscopy was a barrier for them. A man from the urban area said: “Well, [about] the colonoscopy, is the diet that you have to follow before getting tested, which I don’t agree with. Following the diet that one must do before the test it is very difficult for me.” Also, on this regard, a woman from the urban area spoke about being uncomfortable with the preparation process stating: “We have to drink a liquid and that’s what I don’t like.”

#### Perception of Masculinity

Men in both settings agreed that another barrier to having a colonoscopy was the way in which the test was performed. Our participants indicated that they were uncomfortable with the procedure because it required having a tube inserted in their rectum (urban setting: “and he [the doctor] introduced that thing and I said: Hey, what are you doing!”). They indicated that “[…] men have their pride” (urban setting man) and that culturally this type of action is not something a man would approve of. It was also pointed out that in their experience, most healthcare providers that perform this type of procedure are women. Having a woman, especially if they are younger, made them feel embarrassed and uncomfortable. A man from the rural clinic indicated: “Those tests… remember that women always do them [perform the tests]. Women are the ones who do those tests to detect colon cancer, and prostate cancer… I get embarrassed because they ask you to take off your clothes… One gets embarrassed.” Another from the same clinic said: “[…] but I felt a little embarrassed by the girl's age. Do you understand me?”.

#### Transportation Barriers

Mostly participants from the rural area discussed transportation barriers to locations where CRCS is provided. The problem, as described, is that tests such as the colonoscopy are performed in the municipality of Arecibo that is 58.9 km from Castañer and the trip takes approximately 1 h 23 min. To travel from Castañer to Arecibo, participants must take public transportation, which is not very accessible, or pay for private transportation. A woman from the rural setting mentioned: “I do not have a car; most people here do not have a car. We depend on [public transportation] to take us and wait for us. Appointments are distributed in order of arrival, and they start to distribute them at 4 am. Suppose [you] arrive at 9:30 am, by the time you arrive the appointments are too late and you have missed the chance [to be seen]. It's a barrier for us here in Castañer.” A man from the same area said: “I would do it (CRCS), but since I do not have a car or someone that would take me and here they charge $50 to take you…” Furthermore, since the procedure requires a companion (patients are not allowed to drive after the procedure), transportation can be a problem when the person relies on public transportation. A woman from the urban area said: “If you’re going to take the test (colonoscopy) you cannot go by fare, you must go with a person who takes you, remember it's under anesthesia.”

#### Psychosocial Barriers to CRCS

In addition to transportation barriers, participants mentioned laziness, lack of time, and being alone as barriers to CRCS. Regarding the FOBT, a woman from the rural area was asked why she had not gotten the test done yet and she replied: “Well, because I’m lazy.” Another participant also agreed that laziness was a reason to not get the FOBT. A woman from the rural area was asked if she would get a colonoscopy if a doctor recommended it to her, to which she responded: “No because I do not have the time.” Taking into consideration the transportation barriers already reported particularly by patients from the rural area, a male participant was asked if having to be transported to another municipality to do the test was a problem, to which he responded: “Yes, it is a problem because one is alone.” Lastly, a patient from the urban area stated a mobility problem that limits her ability to walk as a barrier to CRCS: “I’m telling you that it will depend on how I am because I have spurs that are developing in one of my feet […].”

#### Financial Burden of CRCS

For participants of both urban and rural areas, not having money for co-payments were mentioned as a barrier to CRCS. In our study, participants were from low-income households and indicated that paying for CRCS could not be doable if uninsured or even if insured because the screening test is not being fully covered by their insurance. A man from the urban clinic said: “If you don’t have insurance, you have to pay a lot of money to get it [colonoscopy] done.” A woman from the rural area mentioned,”[…] another barrier is that we have to pay deductibles [co-payment], and, for example, I have no means to pay deductibles; so most of the others here.“ A woman from the urban area said:”Well, because one does not know if at least the Reforma (Government Health Plan) covers it. [If it’s not covered] I cannot do the test for economic reasons.” Another woman from the rural area explained the complications behind the government health plan: “The doctor has to communicate with the headquarters [of Triple S Reforma] and give my name, addresses and all that stuff to see if they authorize it and, if they don’t authorize it, you can’t get it done.” Females from the urban area also indicated that not knowing if their health insurance would cover the tests was a barrier because they needed to know beforehand if they had to pay for deductibles. One of them said “[…] sometimes you don’t know if that [the test] will be covered. Right now, I wasn’t paying [for the FOBT]; now I have to pay.” When asked if not having health insurance could be a barrier, one of the female participants from the urban area said: “If the health insurance doesn’t cover it [the colonoscopy], it’s probably very costly.” Another statement was also brought up by a male from the urban area regarding the colonoscopy: “When you don’t have health insurance, you have to pay a lot of money to do it [the colonoscopy].”

### Lack of Provider Recommendation

Participants from all settings said that communication with physicians regarding recommending the CRCS test was lacking. Many participants indicated not having received at all recommendation from their physician to get screened. Others shared that they had been advised to get tested, but their physicians did not take the time to explain why they were recommending the screening test and what it was for. A man from the urban area commented about the FOBT: “I understand that one of the things that hinders that first step that one has to take is to identify, what, why, and for what [is CRCS], and doctors do not really explain that. One goes, they ask what your symptomatology is, and based on that symptomatology, they tell you.” Additionally, some participants mentioned that they do not feel comfortable bringing up a conversation about CRC or CRCS to their primary provider as one barrier to CRCS. A woman from the urban area said: "I do not have that confidence. I do not know, because I have only had 2 appointments with that doctor and like, that does not give me confidence. I do not know… I do not talk about anything and he is not very talkative either."

## Discussion

This qualitative study explored knowledge, perception of risk, and barriers that might influence early detection of CRC and CRCS among study participants from FQHCs in Puerto Rico from rural and urban settings. In our study and prior to the brief explanation of CRC and CRCS, participants mentioned that they did not have a clear understanding about CRC and demonstrated very low knowledge about the different CRCS tests as well as the procedures that occur before, during, and after the colonoscopy. While they could not articulate the benefits of CRCS because of low levels of knowledge in the groups, once the moderator described CRC, CRCS, and its benefits, all participants discussed the importance of getting screened. An important observation was a low perceived risk of CRC in this group, which has been prior documented in similar studies across race/ethnic groups in the US [9, 10]. Additionally, participants discussed culturally influenced attitudes such as masculinity, fatalism, and procrastination, and feelings of fear and embarrassment concerning the colonoscopy procedure, fear of test results, and perceived barriers such as lack of time and transportation problems.

Barriers observed in our study have been consistent with those in the literature, particularly among Hispanics [11–13]. As has been previously documented for colonoscopy, and was shown in our focus groups discussion, embarrassment—particularly for men—might deter the opportunity for a timely screening due to men’s sense of their masculinity [8, 14]. Studies of screening for prostate [15] and testicular cancer [16] have shown similar findings. Similar to findings in other studies with Latinos in the USA [8, 12, 17], our participants indicated how the “invasiveness” of the colonoscopy procedure was a deterrent for screening as the procedure was embarrassing, and affected their pride and sense of masculinity. Likewise, having a sense of fatalism is a barrier for CRCS. In our study, we found that both men and women have an understanding that a cancer diagnosis is a death sentence. Other studies with Latinos [8, 12] have also presented similar results; but this belief can also be seen in other populations. In a study among Safety-Net Clinics [18], it was shown that Hispanic and non-Hispanic Black patients have a higher sense of fatalism regarding CRCS when compared to their non-Hispanic White counterparts. It should be noted, however, that the perceptions expressed in these focus groups were related to the fear of finding out one had cancer and the beliefs about the inevitability of death if one had cancer and not a sense of “predeterminism” that if one was supposed to die from cancer they would, regardless of what they did, This is an important distinction from a health promotion perspective since messages designed to influence survivability beliefs are likely more effective than messages to modify ingrained pre-deterministic beliefs [19]. These findings suggest that in order to increase CRCS, culturally tailored messages that offer positive role models of people who have gotten screened, found cancer, and been successfully treated (i.e. testimonials from survivors) are needed and therefore must be developed.

According to the NCCR [20], procrastination is among the top barriers for CRCS among unscreened Hispanic/Latinos. When compared to Vietnamese and Non-Latino White Americans, Walsh et al. (2004) found that Latinos were more likely to delay any CRCS tests (FOBT, sigmoidoscopy, or colonoscopy) if given or referred by a doctor [21]. In PR, other colleagues such as Portilla-Skerrett et al. (2019) found that the barriers reported among Puerto Ricans aged 40 years or older that participated at two cancer awareness events included some participants reporting “laziness” and that they “don’t feel it is necessary” [22].

Participants mentioned that financial burden was another barrier for not adhering to CRCS practices. A qualitative study conducted by Byrd et al. (2019) among Hispanics of El Paso, Texas, researchers identified that the costs of the screening tests were among the barriers mentioned by the participants. The participants indicated the difficulty of finding a health insurance that helps them pay their screening tests and that people would perform the screening tests if they did not have to pay so much [23]. Furthermore, out-of-pocket costs have also been evidenced as a barrier to CRCS, which is something that worried the participants in this study. Perisetti et al. (2018) evidenced that limited financial resources, specifically out-of-pocket costs, led to colorectal cancer non-screening within the past 12 months and their finding was statistically significant (*p* < 0.0001) [24].

There were few differences between rural and urban groups. This finding is similar to those of a study among Hispanic men and women from urban Dauphin county and Juniata county in Pennsylvania, where barriers to CRCS were similar among groups (urban/rural setting) [25]. This lack of difference in our study might be due to the homogeneity in the setting in which our study was conducted (FQHCs), in which services for CRC prevention might be similar across clinics, irrespectively of their geographical variability. There is also vast coverage of medical insurance in our sample, in both rural and urban setting participants. Among the participants in our focus groups, 72% of them are beneficiaries of the PR Government Health Plan. This health insurance covers 93% of the population, since Law 72 establishing the Government Health Insurance Plan was implemented in 1993. This governmental insurance covers several types of CRCS tests; hence, physicians should be informed about this so that they can inform their patients about the different options available for screening.

## Conclusions

In this group of non-adherent Puerto Rican participants aged 50 and older, misconceptions about CRC, screening tests available, and testing procedures were evident. There were few differences in the barriers discussed among participants from rural vs urban settings. Barriers such as lack of provider recommendation was an important factor limiting CRCS among both rural and urban participants. As expected, provider recommendation played an important part regarding willingness and acceptance of the tests. Findings of this study had important implications for the development of outreach and health promotion activities in the island, with the implementation of *¡Salud!, por la Vida* (SPLV), an educational intervention addressed to increase CRCS among Puerto Ricans 50 years or older that receive health care treatment at federally qualified health clinics. By using the systematic framework known as intervention mapping [5], a tailored interactive multimedia intervention was developed for SPLV and is currently being tested in the island to evaluate its effectiveness. The results of this study can inform the development of CRCS promotion interventions including the use of innovative strategies to increase CRCS and reduce CRC-related health disparities.

## Supplementary Information

Below is the link to the electronic supplementary material.

Supplementary file1 (DOCX 17 KB)

Supplementary file2 (DOCX 16 KB)

## Data Availability

Not applicable.
