# Supplementary material for: Knowledge, Attitudes, and Beliefs About Colorectal Cancer Screening in Puerto Rico
Source: J Cancer Educ. 2022 Mar 31;38(2):552–61. doi: 10.1007/s13187-022-02153-z (PMC10102089; doi:10.1007/s13187-022-02153-z)
Supplement: Supplementary file 1 — Supplementary file1 (DOCX 17 KB) [file 13187_2022_2153_MOESM1_ESM.docx]

## **Knowledge, Attitudes, And Beliefs About Colorectal Cancer Screening In Puerto Rico**

J. Cancer Education

## **Colón-López, Vivian, PhD., MPH^1,2^; Valencia-Torres, Ileska M. BS^3^; Ríos, Elsa I., DrPH.^1^; Llavona, Josheili, MS.^4^; Vélez-Álamo, Camille MS^4^, and Fernández, María E. PhD^3^**

^1^ Division of Population Health Sciences, PR Comprehensive Cancer Center, University of Puerto Rico, Medical Sciences Campus, PMB 371 P.O. Box 70344, San Juan, PR 00936-5067

^2^ Health Services Administration, Evaluation Program, Graduate School of Public Health, University of Puerto Rico; PMB 371 P.O. Box 70344, San Juan, PR 00936-5067

^3^ The University of Texas School of Public Health, Center for Health Promotion and Prevention Research, 7000 Fannin St., Suite 2080, Houston, TX 77030

^4^ UPR-MDACC Partnership for Excellence in Cancer Research Program, University of Puerto Rico, PMB 371 P.O. Box 70344, San Juan, PR 00936-5067

**Corresponding Author:** Vivian Colón-López, PhD. Email: [vivian.colon@upr.edu](mailto:vivian.colon@upr.edu)

Division of Population Health Sciences, PR Comprehensive Cancer Center, University of Puerto Rico, Medical Sciences Campus, PMB 371 P.O. Box 70344, San Juan, PR 00936-5067

Phone: (787) 758-2525 ext. 1401 | Fax: (787) 522-3282

**Online Resource 1: Focus groups sociodemographic characteristics (n=50*)**

| **Sociodemographic characteristics** | **Number (%)** |
| --- | --- |
| **Age** **Mean ± SD** | 61.9 ± 6.8 years |
| **Gender** |  |
| Female | 28 (56.0) |
| Male | 22 (44.0) |
| **Civil Status** |  |
| Never married | 12 (24.0) |
| Married or living together | 18 (36.0) |
| Divorced or separated | 14 (28.0) |
| Widower | 6 (12.0) |
| **Education (years)** |  |
| ≤ 12 | 37 (77.1) |
| > 12 | 11 (22.9) |
| **Occupation** |  |
| Unemployed | 38 (76.0) |
| Full time Job | 5 (10.0) |
| Part-time Job | 2 (4.0) |
| Disabled / pensioner | 5 (10.0) |
| **Annual Income** |  |
| Less than $15,000 | 43 (87.8) |
| $15,000 to $34,999 | 6 (12.2) |
| **Health Insurance** |  |
| None | 2 (4.0) |
| Puerto Rico Government Health Plan (Reforma) | 36 (72.0) |
| Private Health Care Plan | 6 (12.0) |
| Medicare | 6 (12.0) |

**50 participants completed the survey*
