# Supplementary material for: Knowledge, Attitudes, and Beliefs About Colorectal Cancer Screening in Puerto Rico
Source: J Cancer Educ. 2022 Mar 31;38(2):552–61. doi: 10.1007/s13187-022-02153-z (PMC10102089; doi:10.1007/s13187-022-02153-z)
Supplement: Supplementary file 2 — Supplementary file2 (DOCX 16 KB) [file 13187_2022_2153_MOESM2_ESM.docx]

## **Knowledge, Attitudes, And Beliefs About Colorectal Cancer Screening In Puerto Rico**

J. Cancer Education

## **Colón-López, Vivian, PhD., MPH^1,2^; Valencia-Torres, Ileska M. BS^3^; Ríos, Elsa I., DrPH.^1^; Llavona, Josheili, MS.^4^; Vélez-Álamo, Camille MS^4^, and Fernández, María E. PhD^3^**

^1^ Division of Population Health Sciences, PR Comprehensive Cancer Center, University of Puerto Rico, Medical Sciences Campus, PMB 371 P.O. Box 70344, San Juan, PR 00936-5067

^2^ Health Services Administration, Evaluation Program, Graduate School of Public Health, University of Puerto Rico; PMB 371 P.O. Box 70344, San Juan, PR 00936-5067

^3^ The University of Texas School of Public Health, Center for Health Promotion and Prevention Research, 7000 Fannin St., Suite 2080, Houston, TX 77030

^4^ UPR-MDACC Partnership for Excellence in Cancer Research Program, University of Puerto Rico, PMB 371 P.O. Box 70344, San Juan, PR 00936-5067

**Corresponding Author:** Vivian Colón-López, PhD. Email: [vivian.colon@upr.edu](mailto:vivian.colon@upr.edu)

Division of Population Health Sciences, PR Comprehensive Cancer Center, University of Puerto Rico, Medical Sciences Campus, PMB 371 P.O. Box 70344, San Juan, PR 00936-5067

Phone: (787) 758-2525 ext. 1401 | Fax: (787) 522-3282

**Online Resource 2: Risk factors and Health behavior (n=50)**

| **Characteristic** | **Number (%)** |
| --- | --- |
| **Alcoholic beverages consumption** |  |
| 7 days a week | 1 (2.0) |
| 14 days in the past 30 days | 1 (2.0) |
| Does not consume alcoholic beverages or hasn’t consumed alcoholic beverages in the last 30 days | 42 (84.0) |
| **Smoke** |  |
| Everyday | 8 (36.4) |
| Some days | 4 (18.2) |
| Doesn’t smoke | 10 (45.5) |
| **Fruit consumption in the past 30 days** |  |
| None | 5 (10.2) |
| < 1 daily portion | 14 (28.6) |
| 1-2 daily portions | 14 (28.6) |
| More than 2 portions-4 daily portions | 13 (26.0) |
| 5 daily portions | 3 (6.1) |
| **Vegetable consumption in the past 30 days** |  |
| None | 3 (6.1) |
| <½ daily portion-1 daily portion | 32 (64.0) |
| 2-4 daily portions | 12 (24.0) |
| 5 or more daily portions | 2 (4.0) |
| **Red meat consumption in the last 4 weeks** |  |
| None | 2 (4.0) |
| ≤1 a week | 36 (72.0) |
| 3-6 a week | 12 (24.0) |
| **CRC screening tests performed** |  |
| Fecal Occult Blood Test (FOBT) | 11 (22.0) |
| Colonoscopy | 3 (6.0) |
